# The Inflammatory Mechanism of Parkinson’s Disease: Gut Microbiota Metabolites Affect the Development of the Disease Through the Gut–Brain Axis

**DOI:** 10.3390/brainsci15020159

**Published:** 2025-02-06

**Authors:** Ai Gao, Jiaqi Lv, Yanwei Su

**Affiliations:** Department of Nursing, Tongji Medical College, Huazhong University of Science and Technology, Wuhan 430030, China; u202212847@hust.edu.cn (A.G.); u202212864@hust.edu.cn (J.L.)

**Keywords:** Parkinson’s disease, gut microbiota dysbiosis, gut–brain axis, gut microbiota metabolites, inflammatory mechanism, treatments

## Abstract

Parkinson’s disease is recognized as the second most prevalent neurodegenerative disorder globally, with its incidence rate projected to increase alongside ongoing population growth. However, the precise etiology of Parkinson’s disease remains elusive. This article explores the inflammatory mechanisms linking gut microbiota to Parkinson’s disease, emphasizing alterations in gut microbiota and their metabolites that influence the disease’s progression through the bidirectional transmission of inflammatory signals along the gut–brain axis. Building on this mechanistic framework, this article further discusses research methodologies and treatment strategies focused on gut microbiota metabolites, including metabolomics detection techniques, animal model investigations, and therapeutic approaches such as dietary interventions, probiotic treatments, and fecal transplantation. Ultimately, this article aims to elucidate the relationship between gut microbiota metabolites and the inflammatory mechanisms underlying Parkinson’s disease, thereby paving the way for novel avenues in the research and treatment of this condition.

## 1. Introduction

Parkinson’s disease (PD) is defined by the clumping and deterioration of α-synuclein (α-syn) within the brain, with its prevalence increasing as individuals age [1]. Nonetheless, the precise origin of PD is still unclear [2]. Research suggests that an imbalance in gut microbiota may play a role in the disease’s advancement through the intricate communication network established by the gut–brain axis, creating a damaging cycle [3,4]. Moreover, although the gut–brain axis is crucial, inflammatory processes are also linked to this phenomenon [5]. For example, studies on PD have indicated that gut microbiota dysbiosis can provoke intestinal inflammation and neuropathy via the gut–brain axis, ultimately facilitating disease progression [6]. Additionally, alterations in metabolite composition due to gut microbiota disruption have been found to influence PD by disrupting the gut–brain axis’s two-way communication loop [7]. For instance, an analysis of a fecal sample from a patient with PD showed a reduction in metabolites produced by gut microbiota, particularly short-chain fatty acids (SCFAs). Researchers hypothesize that this decline may be associated with inflammation in either the gastrointestinal or central nervous system, which, as noted earlier, may affect the trajectory of PD [8].

This review intends to examine the inflammatory pathways that connect the dysbiosis of gut microbiota to PD, emphasizing the importance of the metabolites that play a role in these mechanisms. Building on this foundation, we point out the research techniques and treatment methods based on metabolites, hoping to promote the progress of gut microbiota metabolites in the treatment of PD.

## 2. Dysbiosis of Gut Microbiota and Parkinson’s Disease

### 2.1. The Symptoms of Parkinson’s Disease

It is widely recognized that the characteristics of PD extend beyond its motor symptoms to encompass various non-motor symptoms (NMSs). Motor symptoms are the hallmark features of PD, primarily manifested as bradykinesia, muscle rigidity, resting tremors, and postural and gait disturbances [9]. Empirical clinical observations have identified two main subtypes: tremor-predominant PD (characterized by a relative absence of other motor symptoms) and NMS PD (which includes phenotypes such as motor stiffness syndrome and postural instability gait disorders) [10]. NMSs often emerge during the pre-motor phase and significantly impact the quality of life for individuals with PD, including gastrointestinal disorders, pain, and mental health issues. Notably, gastrointestinal dysfunction can manifest as constipation, impaired gastric emptying [11], alterations in gut microbiota, and increased intestinal permeability [12]. Approximately 40% to 85% of PD patients experience pain [13], which can include various types such as visceral and neuropathic pain [14]. The mechanisms underlying pain in PD involve structures such as the substantia nigra and basal ganglia, as well as α-syn infiltration [15]. Furthermore, 35% of PD patients are affected by depression, while 40% experience anxiety disorders [16]. These mental health issues are associated with changes in the neurotransmitter system [17] and the overexpression of α-syn [18].

The treatment and management of motor symptoms in Parkinson’s patients primarily involve pharmacological therapies that enhance dopamine concentrations in the brain or stimulate dopamine receptors [9]. Additionally, rehabilitation strategies, such as PD multimodal comprehensive therapy (PD-MCT) [19], play a crucial role. Regarding the clinical management of NMSs, gastrointestinal issues are prevalent among PD patients, with alterations in the gut microbiome, small intestinal bacterial overgrowth (SIBO), and Helicobacter pylori infections common. Eradicating these infections can lead to symptom improvement [20]. In relation to pain, the gut microbiota may influence pain pathways in PD patients through various mechanisms [21]. Furthermore, the microbiota is linked to anxiety and depressive behaviors, suggesting that manipulating the microbiota could help alleviate emotional disorders associated with PD. Probiotics used to modify intestinal bacteria have shown potential in affecting CNS function, enhancing gastrointestinal symptoms, and improving emotional and pain scores in PD patients [22,23]. This indicates that targeting the intestinal barrier function may represent a novel approach for prevention and treatment [24]. Future research on PD should consider evaluating gut microbiota as an early biomarker, and preclinical studies should investigate its role in the disease’s development and progression.

### 2.2. Dysbiosis of Gut Microbiota in Parkinson’s Disease

The microbiota exists both on the human body’s surface and inside its various cavities, and those that reside in the guts are commonly known as the gut microbiota. Alterations in the makeup of these microbial populations may result in diseases impacting various bodily systems. Consequently, they have attracted considerable interest in extensive research concerning chronic diseases [25,26].

Evidence indicates a relationship between gut microbiota dysbiosis and the development of PD. Specifically, research indicates that there was an increase in the populations of Ackermanella, Salmonella, and Lactobacillaceae in the PD, whereas the populations of Roseburia, Faecalibacterium, and Achromobacteraceae saw a decline [27]. Moreover, an additional study found that those suffering from PD showed diminished levels of Enterococciaceae in comparison to the control group [28]. Nonetheless, the alterations in gut microbiota composition highlighted by these investigations are not comprehensive, signaling a necessity for more research.

## 3. Gut–Brain Axis

The gut–brain axis serves as a link between the gut and the brain, demonstrating a significant role in the development of various neurological disorders [29,30,31,32,33]. Notably, an imbalance in gut microbiota can influence PD via the two-way communication capabilities of the gut–brain axis [34].

### 3.1. Anatomical Pathway

Recent research has emphasized two main mechanisms through which signals originating from the gut microbiota communicate with the brain through the gut–brain axis: (1) via the nervous system, in which the enteric nervous system and the vagus nerve work together; and (2) via the circulatory system, which mainly aids in the transportation of different chemical signals, such as neurotransmitters and cytokines [35].

#### 3.1.1. The Neural Pathway

The gut–brain axis enables two-way communication using two unique neuroanatomical pathways. The initial pathway is the enteric nervous system, adept at sensing external stimuli. The second pathway is the vagus nerve, which forms synaptic links with neurons responsible for sensory and motor activities in the enteric nervous system [36,37,38]. These synaptic links are crucial for efficient two-way communication, allowing neural signals from the external environment to travel to the central nervous system through the autonomic nervous system and then sending signals back [39].

#### 3.1.2. Systemic Circulation Pathway

The pathway of systemic circulation mainly enables the movement of particular chemicals between the brain and the gut [40]. The immune and endocrine systems largely regulate the formation of these chemicals, impacting brain function through the creation of these compounds [41]. In addition, this communication is reciprocal, permitting the flow of signals from the gut to the brain and the other way around, thus creating an ongoing cycle of influence [42].

### 3.2. The Bidirectional Transmission of Inflammatory Signals

Due to the disturbance of the intestinal barrier and the inflammation of the gut resulting from dysbiosis in gut microbiota, the bidirectional transmission of inflammatory signals along the gut–brain axis has been activated [43].

Specifically, the first mechanism involves the neural pathway. Within the ascending neural pathway, the intestinal barrier serves not only to block pathogens from infiltrating the bloodstream but also to uphold the stability of the intestinal microenvironment [44]. If the intestinal barrier is weakened due to dysbiosis, pathogens may breach the bloodstream, causing immune and inflammatory cells in the intestine to release inflammatory mediators, which results in intestinal inflammation [45,46]. Moreover, it has been proposed that inflammation in the guts induces oxidative stress and promotes the misaggregation of α-syn within intestinal neurons. The misaggregated α-syn proteins could potentially travel to the brain via the vagus nerve pathway, thereby affecting the distribution of α-syn within the brain and playing a role in the advancement of PD [47]. In the nerve pathway that descends, predominantly cholinergic vagal efferent fibers stimulate enteric neurons, leading to the release of neurotransmitters that impede the generation of specific inflammatory agents, which in turn affect intestinal inflammation [48,49].

**Figure 1:**
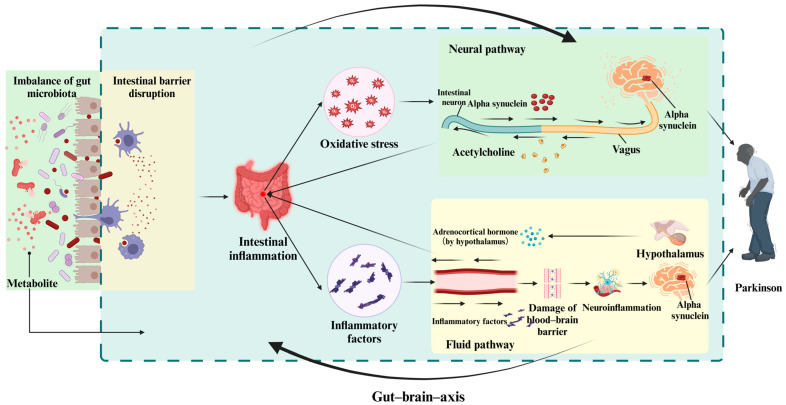
Imbalances in gut microbiota can compromise the integrity of the intestinal barrier, resulting in intestinal inflammation. The inflammatory signals generated can influence the aggregation and deformation of alpha-synuclein (a-syn) in the brain via neural and humoral pathways. Due to the bidirectional communication between the gut and the brain, the brain can also modulate intestinal inflammation through these same pathways. This figure is prepared in https://BioRender.com (accessed on 29 January 2025).

Conversely, the systemic circulation pathway is also affected. In the gut–brain ascending pathway, the intestinal barrier can become compromised, resulting in inflammation of the intestine. As a result, inflammatory substances or infectious agents could access the brain via the systemic circulation, subsequently enhancing the permeability of the blood–brain barrier (BBB) and triggering the activation of microglial cells [50]. This stimulation initiates oxidative stress and worsens neuroinflammation [51,52]. Recent research suggests that factors like oxidative stress and neuroinflammation play critical roles in the advancement of PD [53]. In the descending pathway, when the central nervous system (CNS) receives an integrated response to intestinal inflammation, it can impact intestinal integrity through the hypothalamic–pituitary–adrenal axis (HPA), further affecting intestinal inflammation [54,55] (Figure 1).

## 4. Gut Metabolites Affect Gut–Brain Axis

### 4.1. Key Links of the Bidirectional Inflammatory Signal Transmission

The integrity of gut and blood–brain barrier, as well as the degree of inflammation present, plays a vital role in the two-way communication of inflammatory signals between these systems. Changes in these aspects can affect how inflammatory signals are exchanged within the gut–brain axis [56].

Specifically, in the gut, the impairment of the intestinal barrier can result in inflammation within the gut, which may change the inflammatory signals traveling from the gut to the brain, thus affecting the degree of inflammation present in the brain. On the other hand, the brain can react to the signals it receives from the intestine by modifying the inflammatory messages that move from the brain to the gut, establishing a feedback loop that continues [57]. Additionally, within the brain, the compromise of the BBB heightens the risk of neuroinflammation. Considering the gut–brain axis, variations in inflammation levels can alter the inflammatory signals in the pathway descending from the gut–brain axis, which, in turn, can influence the inflammation levels within the gut. This change subsequently impacts the brain through the ascending pathway of the gut–brain axis, ultimately leading to the development of a cyclical relationship [58].

In this bidirectional communication process, inflammation that starts in the intestinal area may be identified as peripheral inflammation. Typical accompanying symptoms encompass gastrointestinal issues like abdominal discomfort, diarrhea, and nausea, in addition to systemic signs such as fever, exhaustion, and weight loss. In contrast, inflammation that arises from the brain can be defined as central inflammation, presenting symptoms that include difficulties with concentration, memory impairment, emotional swings, depression, anxiety, and seizures, alongside muscle cramps. Despite the different functions that peripheral and central inflammation fulfill in their specific regions, they both ultimately affect the progression of PD through the bidirectional interactions of the gut–brain axis [55].

### 4.2. Metabolites Affect the Bidirectional Transmission of Inflammatory Signals

Metabolites produced by gut microbiota can be divided into three categories depending on their sources and the processes of synthesis involved [59]. The primary sites where inflammatory reactions occur vary due to the distinct locations influenced by different metabolites. Considering the variations in symptoms between central and peripheral inflammation discussed earlier, there are also differences in how metabolites impact symptoms of PD via inflammatory mechanisms [55].

#### 4.2.1. Metabolites Resulting from Dietary Elements

**Figure 2:**
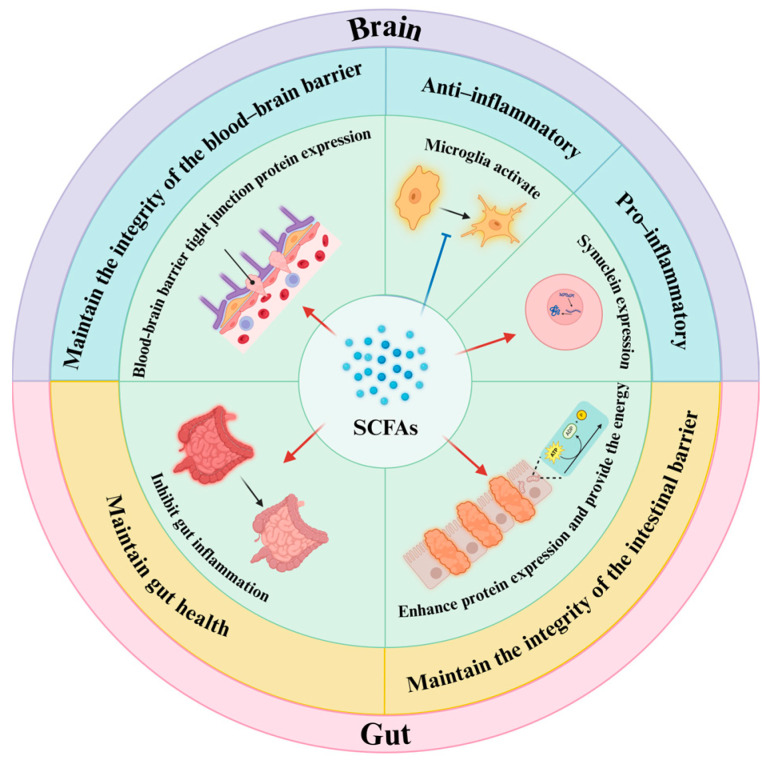
Short-chain fatty acids (SCFAs) are among the most significant metabolites produced by gut microbiota. They play a crucial role in the inflammatory mechanisms associated with Parkinson’s disease by protecting the gut barrier and the blood–brain barrier, inhibiting intestinal inflammation, and exerting both positive and negative effects on neuroinflammation. This figure is prepared in https://BioRender.com (accessed on 29 January 2025).

SCFAs: SCFAs are a category of saturated fatty acids, and they are particularly acknowledged for their impact of the inflammatory processes linked to PD through the modulation of the gut–brain axis [60,61,62]. A recent study suggested that SCFAs could influence the brain by modifying the stability of the BBB through two main mechanisms. First, increasing the production of tight junction proteins may bolster the maintenance of the integrity of the BBB. A study conducted with a mouse model, for instance, showed that administering butyrate led to elevated levels of tight junction proteins, such as occludins and ZO-1 [63]. Secondly, Hoyles et al. [64] demonstrated that SCFAs can protect the BBB by modulating nonspecific inflammatory responses and oxidative stress. Moreover, several studies have found that SCFAs can also have a hold on the brain through both positive and potentially negative effects in regulating microglia and neuroinflammation. Liu and associates [65,66] suggested that SCFAs might possess the ability to reduce the activation of microglia and inhibit the generation of inflammatory cytokines, potentially leading to an anti-inflammatory effect and a protective role for neural function. Conversely, Duan et al. [67] proposed that SCFAs could exacerbate neuroinflammation by promoting the overexpression of α-syn. In addition, studies suggest that SCFAs could have an impact on the gut by influencing its barrier via two different mechanisms [68]: firstly, SCFAs could provide energy to colon epithelial cells, reduce cell apoptosis, and subsequently protect the intestinal barrier; and, secondly, SCFAs have the ability to increase the expression of proteins associated with intestinal tight junctions, thereby further improving the protective function of the intestinal barrier. Moreover, an observation implied that SCFAs could also affect gut function by contributing to the reduction in intestinal inflammation. Specifically, in a mouse model examining intestinal inflammation induced by Salmonella, researchers discovered an inverse relationship between the levels of the inflammatory marker lipid carrier protein-2 and the overall concentration of SCFAs [69] (Figure 2).

Tryptophan metabolism: Kynurenic acid (KYNA), anthranilic acid (AA), quinolinic acid (QA) [70,71,72], 5-hydroxytryptamine (5-HT) [73], as well as indole and its derivatives [74], represent final metabolic products. To begin with, the metabolite KYNA acts as a significant non-competitive antagonist of the N-methyl-D-aspartate (NMDA) glutamate receptor, and it also exhibits antioxidant characteristics that could assist in reducing neuroinflammation [71]. Additionally, QA may influence the inflammatory signaling pathway through tryptophan metabolites produced by the gut microbiota. This influence may help reduce oxidative stress and neuroinflammation in the mice on a high-fat diet (HFD) [75]. Furthermore, multiple research investigations have indicated that the 5-HT plays a role in gut through maintaining intestinal homeostasis [76]. In addition, the indole and its derivatives could also maintain immune homeostasis and intestinal barrier function. For example, a research investigation indicated that the gestational X receptor was influenced by the combination of index and propofol; this may improve the effectiveness of the intestinal mucosal barrier, lower the permeability of the intestinal lining, and reduce the occurrence of intestinal inflammation [77].

#### 4.2.2. Metabolites Produced by the Host but Modified by Gut Microbiota

Bile acids (BAs): BAs originate from cholesterol within the liver through different metabolic routes. After their production, they undergo conjugation with either taurine or glycine, subsequently entering the bile before reaching the gut, where gut microbiota transforms them into secondary BAs [78]. The brain, a crucial part of the nervous system, holds nearly all varieties of bile acid receptors, facilitating bile acids having the potential to affect brain function through their interaction with these receptors [79]. Among these receptors, Takeda G-protein-coupled receptor 5 (TGR5) has emerged as a most prominent one, capable of interacting with the endogenous agonist tauroursodeoxycholic acid (TUDCA) and the semi-synthetic bile acid INT777 [80]. This interaction contributes to neuroprotection and demonstrates anti-inflammatory properties [81]. BAs may also have a significant impact on gut function [82,83]. In particular, they help maintain the integrity of the mucus layer and tight junctions by modulating the farnesoid X receptor (FXR) and TGR5, enabling immune cells to perform anti-inflammatory functions and regulatory roles associated with immune response [84,85].

#### 4.2.3. Metabolites Synthesized Autonomously by the Gut Microbiota

Polyamines: Compounds known as polyamines, which include putrescine, spermidine, and spermine, are produced by the gut microbiota [86,87]. They are primarily synthesized from ornithine and arginine, indicating that ornithine decarboxylase and arginine decarboxylase play crucial roles in the synthesis and metabolism of polyamines [88]. In an animal study, researchers discovered that dietary spermidine can penetrate the brain primarily by enhancing cellular autophagy and mitophagy, thereby improving mitochondrial function and facilitating cognitive recovery in PD [89]. Moreover, some compounds known as polyamines, could contribute to sustaining the intestinal barrier’s integrity, which is indicated by lower concentrations of fecal metabolites found in individuals with PD [90]. In a mouse experiment, mice treated with polyamine inhibitors exhibited reduced epithelial cell proliferation and an increased risk of developing colitis compared to the control group that did not receive inhibitors. These findings suggest that polyamines play a crucial role in the proliferation of intestinal epithelial cells and in the regulation of inflammation levels [91].

Vitamin: The gut microbiota can synthesize various B vitamins, and the majority of them are generated in the large intestine prior to their absorption [92]. Numerous studies have verified the neuroprotective properties of B vitamins. For instance, the concentration of inflammatory cytokines in dendritic cells (DCs) grown in a B7(biotin)-deficient medium was observed to be increased [93]. Additionally, mice fed a diet high in saturated fatty acids exhibited impairments in the BBB and indications of neuroinflammation, which improved following treatment with nicotinate or nicotinamide [94]. In addition, one study showed that the mice lacking the sodium-dependent multivitamin transporter (SMVT), which is capable of not only transporting B5 (pantothenic acid) but also B7 (biotinin), resulted in severe inflammation and increased intestinal permeability, indicating that one or both of these vitamins are crucial for maintaining intestinal homeostasis [95].

Lipids: Lipids are compounds that do not dissolve in water but are soluble in nonpolar organic solvents. They can be divided into eight unique subclasses: fatty acyls, glycerides, glycerophospholipids, sphingolipids, sterols, acyls, glycolipids, and polyketides. Not only do they function as energy reserves, but they also play a crucial role as essential components of cells [96]. In the nervous system, lipids can exert both beneficial and adverse effects. On one hand, polyunsaturated fatty acids [97], monoacylglycerol, and phosphatidylserine [98] can provide neuroprotection through various mechanisms, while triacylglycerol [99] may directly reduce inflammation. Conversely, saturated fatty acids [100] and diacylglycerol [101] can contribute to neuroinflammation, subsequently impacting brain function. Moreover, Tong et al. [102] found multiple associations between lipids and PD, indicating that, in the gut, the role of lipids is predominantly negative. For example, a high intake of fat can result in heightened intestinal permeability, and specific long-chain fatty acids might also affect the control of the tight junction barrier [103].

Branched-Chain Amino Acids (BCAAs): BCAAs play an important role in human health and are commonly used as beneficial supplements. They are widely utilized in food additives, pharmaceuticals, and nutritional fortifiers [104]. In one Mendelian randomization study, researchers examined the relationship between BCAAs as a potential causal factor and PD as the outcome. The findings indicated that BCAA supplementation exerts a protective effect against PD. And the reduction in brain inflammation may be a significant contributing factor in this protective mechanism, when considered by previous studies [105]. Another study employed the classical oral rotenone mouse model, which mimicked the pathological manifestations of PD. Following the oral intake of rotenone, elevations in the levels of TNF-α, IL-1β, and IL-6 were noted in the colon. However, the inclusion of BCAAs resulted in a reduction in the inflammatory response in the gastrointestinal tract [106]. This finding suggests that BCAAs may influence brain function by modulating inflammation levels in the gut.

Neurotransmitters: The gut microbiota produces various neuroactive compounds, including neurotransmitters and their precursors [107]. Compounds such as glutamate, gamma-aminobutyric acid (GABA), and acetylcholine cannot cross the BBB and might influence the gut with the help of the enteric nervous system [108,109]. In a study focusing on glutamate, research by Li et al. [110] demonstrated that glutamatergic neurons in mice can affect motor symptoms related to PD, indicating a potential role of glutamate in the progression of PD. Conversely, certain neurotransmitter precursors, like hydroxytryptamine (which is a precursor to serotonin), are able to traverse the BBB. These precursors move through the bloodstream to reach the brain, where they carry out their functions [111]. A human study analyzing plasma and cerebrospinal fluid samples from both patients and controls revealed altered levels of canine urine metabolites in the peripheral or central nervous system of individuals with PD. This finding suggests that the neurotransmitter precursor tryptophan may also be implicated in the pathogenesis of PD [112].

## 5. Research Methods and Techniques Based on the Metabolites of Gut Microbiota

### 5.1. Detection Method of Gut Microbiota Metabolites

Metabolomics serves as a widely used approach for identifying metabolites generated by gut microbiota. As the most downstream area of systems biology, metabolomics is directly related to biological phenotypes and provides distinct benefits compared to other upstream omics. On one side, minor alterations in genomic or proteomic functions can be escalated at the metabolic level, making detection more straightforward. In contrast, the amount of metabolites found in organisms is significantly lower compared to the number of genes and proteins. In addition, many common metabolites exhibit similarities across different organisms, which facilitates metabolomic research. Additionally, other omics are unable to dynamically analyze processes occurring within an organism. Given the temporal sensitivity of certain living organisms, other omics also fall short in individual diagnostic capability compared to metabolomics. These advantages broaden the application of metabolomics in detecting metabolites of gut microbiota [113,114,115]. Metabolomic analysis is primarily categorized into two approaches: targeted and non-targeted methods. The goal of non-targeted metabolomics is to investigate unknown metabolites without the need for prior identification, facilitating the discovery of metabolic information that may not have been recognized before. In contrast, targeted methods concentrate on specific known metabolites, which are often employed for metabolite quantification and typically exhibit higher sensitivity and reproducibility [114]. In metabolomics research, the key methods employed for the separation of metabolites include Liquid Chromatography (LC) and Gas Chromatography (GC). At the same time, detection is predominantly conducted using Nuclear Magnetic Resonance (NMR) and Mass Spectrometry (MS). NMR can be applied as an independent technique, bypassing the prior analyte isolation step, whereas MS is frequently used in conjunction with LC or GC [116].

In a metabolome association study examining PD across two distinct populations of patients and controls, changes in metabolite levels linked to inflammatory mechanisms in PD were identified. In particular, p-cresol, a uremic toxin originating from external sources and mainly generated by gut microbiota, has been demonstrated in laboratory settings to trigger oxidative stress and inflammatory responses, thus playing a role in the inflammatory mechanisms linked to PD. Additionally, itaconate, known for its anti-inflammatory properties, is found at low levels in PD patients, coinciding with significant inflammation. During this inflammatory response, amino acid levels become dysregulated and are closely associated with these inflammatory mechanisms. For instance, the level of pyroglutamate (PGA), an endogenous metabolite of glutamate, increases in patients, which may be linked to disruptions in glutathione metabolism. Consequently, elevated systemic concentrations of PGA could suggest an improvement in glutathione metabolism aimed at addressing the oxidative stress and inflammatory conditions related to PD. Finally, this study indicates that lipid metabolites are also implicated in PD. In this context, the metabolism of glycerophospholipids, glycosphingolipids, and sphingolipids is found to be enriched, with the pathogenesis of PD related to lipid metabolism involving oxidative stress and inflammation. This indicates that lipids could be involved in the inflammatory processes impacting individuals with PD [117].

In addition to the aforementioned interpretation of metabolomics applications in gut microbiota metabolites, there are notable drawbacks. Metabolomics is a relatively young discipline with limited coverage compared to upstream disciplines such as genomics and transcriptomics, which ultimately leads to challenges in the interpretation of results. Therefore, it is advisable to combine metabolomics with these upstream disciplines, employing a multi-omics approach to study gut microbiota metabolites [118]. This integrative strategy may facilitate the discovery of novel biomarkers or pathological pathways, thereby accelerating the development of new drugs for improved therapeutic efficacy [119,120].

### 5.2. Animal Models

Animal models are often used to study the metabolites produced by gut microbiota and the mechanisms that lead to inflammation related to PD. In a study conducted by Sampson TR and colleagues [121], the researchers utilized a mouse model of PD, developed using a heterozygous approach, to explore the relationship between motor symptoms and SCFAs. Following the administration of these fatty acids, the activation of glial cells and neuroinflammatory responses were noted in the mice. Subsequent treatment with antibiotics resulted in reduced neuroinflammation and an improvement in motor symptoms. These results indicate that SCFAs could aid the advancement of PD via multiple inflammatory processes, such as the activation of glial cells in the brain and the enhancement of neuroinflammation. This experiment allows for a focus on mouse PD models induced through transgenic methods. In clinical studies, it is common to induce animals into a pathological state resembling PD to enhance the relevance of the findings. Currently, there are two primary methods for mimicking PD pathology: the toxin induction model and the genetic model [122]. The toxin-induced model primarily affects PD development [123] by inhibiting respiratory complex I in nerve cells, while the genetic model is based on specific species or in vitro models [124].

Based on various imitation methods for PD pathology, study subjects can be categorized into three groups. The first group comprises rodents, such as mice and similar species. These animals are moderate in size, low in cost, present minimal ethical concerns, and exhibit anatomical and clinical symptoms comparable to those of PD patients. Consequently, they are extensively utilized in neurotoxin and genetic models. The second group includes other organisms, such as zebrafish, Caenorhabditis elegans, and Drosophila, among others. These species possess simpler structures and readily available genomic information, making them popular choices for transgenic models. The third group consists of primates, which, despite their potential applications, are not widely used due to their high cost and ethical implications [125]. Given this context, the study of gut microbiota metabolites and the mechanisms underlying Parkinson’s inflammation faces several urgent challenges, even with the advent of new technologies. Addressing these issues is expected to enhance the scope of experimental research.

## 6. Treatment Methods for Parkinson’s Disease

### 6.1. Therapeutic Prospects Based on the Metabolites of Gut Microbiota

Dietary habits, the use of probiotics, and fecal transplants are among the top three strategies to address PD effectively. These methods aid in optimizing the gut microbiome by boosting the levels of advantageous bacteria, reducing the number of detrimental bacteria, and modifying the production of metabolites. As a result, they enhance the functionality of both the intestinal barrier and the blood–brain barrier, mitigate inflammation, and ultimately support positive therapeutic results in patients with PD.

#### 6.1.1. Dietary Intervention

Ross FC et al. [126] proposed that diet can either promote or inhibit immune activation in the gut through metabolites produced by the gut microbiota, thereby affecting neuroinflammatory processes in the brain and ultimately influencing the progression of PD. Specifically, different dietary patterns impact the levels of gut microbiota metabolites in distinct ways. The Mediterranean diet, a plant-based diet rich in carotenoids, omega-3 fatty acids (ω-3 FAs), polyphenols, vitamins, and dietary fiber [127], is associated with a relatively high bacterial abundance. This diet utilizes fiber as an energy source, promoting the production of SCFAs. In contrast, the Western diet, characterized by high caloric intake, high fat, and high protein content, typically lacks fiber, vitamins, and minerals, which is often linked to a decrease in SCFA production [128,129]. High-fiber and plant-based diets, which are predominantly composed of vegetables and fruits, can alter the composition of the gut microbiota by increasing the abundance of beneficial bacteria. Complex carbohydrates, such as dietary fiber, are fermented by colonic microorganisms, resulting in SCFA production [126,130,131]. The fasting-mimicking diet is a dietary plan that involves long-term fasting, specifically a very low-calorie diet for three consecutive days every other week [132]. This diet has been reported to reduce motor impairment and neuroinflammation in PD mice, improve the composition of the gut microbiome, and increase SCFA levels [133]. The Western diet exacerbates neuroinflammation in Parkinson’s disease (PD) through the microbiota–gut–brain axis, whereas the Mediterranean diet has been shown to reduce neuroinflammation [127]. The ketogenic diet, characterized by a high proportion of fats, a low proportion of carbohydrates, and balanced amounts of protein and other nutrients, can alter the composition of gut microbiota. Specifically, it increases the abundance of Prevotella while decreasing Enterobacteriaceae [134,135]. This shift ultimately inhibits microglial activation and reduces the secretion of pro-inflammatory cytokines in the substantia nigra induced by MPTP, leading to significant improvements in motor dysfunction [136]. Ketone bodies produced during the ketogenic diet, such as β-hydroxybutyrate (β-HB), have been found to mitigate mitochondrial and synaptic dysfunction, oxidative stress, and neuroinflammation, thereby exhibiting neuroprotective effects [137]. Research conducted by CHUC et al. [127] supports these findings, indicating that dietary patterns can influence metabolite composition and subsequently affect the inflammatory mechanisms associated with PD.

#### 6.1.2. Probiotic Intervention to Regulate Metabolites

Probiotics can influence PD through various mechanisms, one of the most significant being the modulation of metabolite production, which in turn affects the inflammatory processes associated with PD [138]. Important metabolites associated with PD comprise SCFAs like acetate [139], propionate [140], and butyrate [141]. Furthermore, probiotics have the ability to generate metabolites of tryptophan [142], as well as primary and secondary bile acids [143,144]. In a mouse study, researchers discovered that the probiotic Bifidobacterium breve CCFM1067 altered the SCFA composition in mice suffering from PD, reduced the expression of proteins and genes associated with inflammation, diminished inflammation in the gut and brain, and enhanced anti-inflammatory responses. As a result, the researchers suggest that the rise in SCFAs could contribute to the anti-inflammatory properties of B. breve CCFM1067 in relation to the gut or brain in the framework of PD [145].

#### 6.1.3. Fecal Transplantation

**Figure 3:**
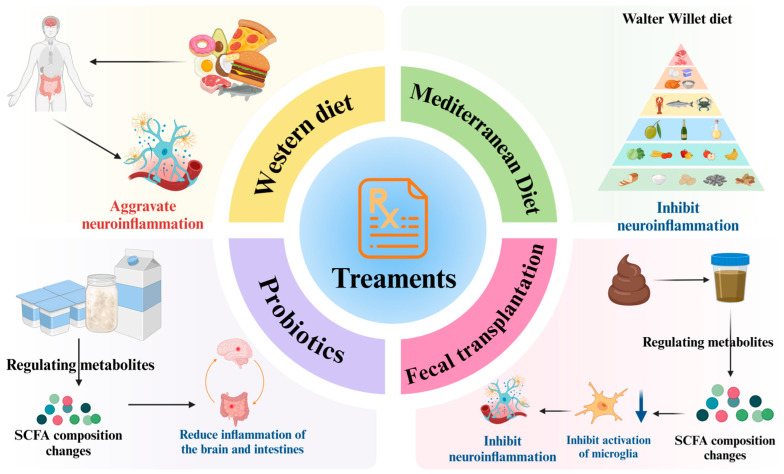
Therapeutic approach based on gut microbiota metabolites: (1) Dietary interventions regulate metabolites: Different dietary patterns can have distinct effects on gut microbiota metabolites, which subsequently influence intestinal inflammation and, ultimately, Parkinson’s disease. For instance, the reduction in short-chain fatty acids (SCFAs) production associated with Western diets exacerbates neuroinflammation, whereas the increased production of SCFAs in the Mediterranean diet can mitigate neuroinflammation. (2) Probiotic interventions regulate metabolites: Probiotics can modify the composition of SCFAs, inhibit the expression of pro-inflammatory genes, and reduce inflammation in both the gut and brain. (3) Fecal transplantation interventions regulate metabolites: Fecal transplantation alters the composition of SCFAs, diminishes the activation of microglia, and inhibits neuroinflammation. This figure is prepared in https://BioRender.com (accessed on 29 January 2025).

Fecal transplants could improve the equilibrium of gut microbiota and aid in certain conditions, including PD. The suggested ways of functioning involve safeguarding the intestinal barrier, decreasing inflammation within the intestines, alleviating damage to the BBB, and suppressing neuroinflammation [138]. Although fecal transplantation is considered an emerging technology in recent years, its origins date back several millennia. For instance, approximately 3000 years ago in China, around 400 BC, a mixture of fresh feces and water was employed to treat food poisoning and diarrhea [146]. In a specific study, scientists examined the fecal concentrations of SCFAs in mice suffering from PD, finding a significant increase in the levels of acetic acid, which showed the greatest change. Upon administering fecal transplantation to the mice, researchers observed a reduction in fecal acetate concentrations in PD mice. Moreover, In mice with PD, the activation of astrocytes and microglia was noted, but this activation subsequently reduced following the fecal transplantation procedure. Integrating findings from previous studies, it appears that SCFAs may promote microglia-mediated neuroinflammation; consequently, fecal transplantation could provide a protective influence in mice with PD by reducing the levels of these fatty acids and suppressing neuroinflammation [147] (Figure 3).

### 6.2. Treatment Methods Based on Parkinson’s Comorbidity Patterns

PD is frequently associated with various comorbid symptoms, particularly those related to certain central nervous system disorders and gastrointestinal conditions. The manifestation of these symptoms may be intricately linked to the pathogenesis of PD. Investigating the emergence of these symptoms enhances our understanding of the underlying mechanisms of PD, offering novel insights and approaches for its diagnosis and treatment.

#### 6.2.1. Parkinson’s Disease and Cognitive Impairment of the Nervous System

PD is a heterogeneous condition that not only results in motor dysfunction but also leads to various deficits in cognition, mental state, autonomic nervous system function, and sensation. Approximately 60% to 80% of patients experience cognitive impairment (CI), with cognitive decline often preceding the onset of motor symptoms [148]. The gut microbiota plays a significant role in the pathogenesis of PD, suggesting that it may serve as a potential diagnostic target for PD-related CI, which is crucial for developing effective treatment strategies. The primary types of CI observed in PD include executive dysfunction, declines in attention and memory, visuospatial impairments, and language deficits [149]. This cognitive decline can progress from mild impairment at the onset of PD to dementia in the advanced stages [150,151,152]. Notably, patients may exhibit cognitive dysfunction even in the early stages of the disease or prior to diagnosis [150]. Currently, the treatment of PD is primarily symptomatic. Levodopa and other medications can alleviate early motor symptoms, while NMS necessitate the use of non-dopaminergic drugs. Additionally, adjuvant therapies, such as rehabilitation, are recommended. However, there are currently no methods available to cure or slow the progression of PD and CI [149]. The treatment of cognitive and neuropsychiatric disorders associated with PD has not advanced significantly, and the mechanisms underlying cognitive decline remain unclear. Reduced synaptic connections and dysfunction of the dopaminergic system may represent critical targets for treatment [153]. While dopaminergic drugs can have cognitive side effects [154], acetylcholinesterase inhibitors have the potential to enhance cognition without adversely affecting motor function. Some medications address PD-related conditions such as depression and hallucinations, although their safety remains questionable [155]. Non-motor symptoms have gained attention due to their substantial impact on patients’ quality of life. Currently, the effects of exercise, cognitive training, and neuromodulation are being actively investigated. Various neuroprotective treatments are undergoing preclinical testing. Furthermore, non-drug interventions, such as deep-brain stimulation (DBS) and gene therapy, are considered effective alternatives to traditional pharmacological treatments. A multidisciplinary approach to managing cognitive decline may enhance the quality of life for patients [156].

#### 6.2.2. Parkinson’s Disease and Gastrointestinal Disorders

In the past decade, significant progress has been made in research concerning gastrointestinal dysfunction in Parkinson’s disease. The gastrointestinal system not only suffers from impaired motor and autonomic nerve functions but also affects the absorption of anti-Parkinson’s medications and plays an active role in the pathophysiological changes associated with motor fluctuations. Evidence indicates that its pathophysiology is closely related to the pathogenesis of Parkinson’s disease [157,158]. The enteric nervous system may serve as a conduit for the spread of α-synuclein, leading to degeneration of the central nervous system [159]. Additionally, it impacts clinical symptoms such as oral difficulties, swallowing problems, and constipation [160]. In light of this, it is crucial to comprehensively assess and treat gastrointestinal dysfunctions in patients. The hypothesis that Parkinson’s disease originates from the gut has garnered significant attention. Although some reports suggest that there is no neuronal loss in the enteric nervous system of patients, the distribution of synucleinopathy is associated with gastrointestinal symptoms. The causal relationship between these factors and the impact of pathological α-synuclein on gastrointestinal function remain to be studied [161]. Patients with Parkinson’s disease frequently experience a range of gastrointestinal issues, including dental problems [162], sialorrhea [163], taste impairment [164], dysphagia [165], malnutrition [166], impaired gastric emptying [20], Helicobacter pylori infection [167], SIBO [168], and constipation [169]. Various treatment approaches are employed to address these issues, such as dental implants, behavioral and pharmacological interventions [170,171], clinical assessments coupled with corresponding training [172], nutritional interventions [173], and a variety of detection methods. Furthermore, treatment options that circumvent the gastrointestinal tract have been developed to mitigate the effects of its dysfunction on motor fluctuations and movement disorders [160].

## 7. Atypical Parkinson’s Syndrome

We have summarized PD from the perspectives of its mechanisms and treatments. Furthermore, atypical Parkinson’s syndrome (APS), which encompasses a group of diseases exhibiting Parkinson’s disease-like symptoms but with distinct clinical manifestations, merits additional exploration. APS can be categorized into alpha-synucleinopathy and tau proteinopathy based on pathological types. The former includes Lewy body dementia (DLB) and multiple system atrophy (MSA), while the latter comprises progressive supranuclear palsy (PSP) and cortical basal ganglia degeneration (CBD) [174].

Research indicates that gut microbiota plays a significant role in the development of PD. Similarly, alterations in gut microbiota are also observed in APS [148]. An observational study [175] separately examined the changes in gut microbiota in the PD and APS groups compared to a control group. The findings revealed that, in the PD group, the abundance of Trichomycteridae decreased, while the reductions in Lachnospiraceae and Lactobacillus, along with the increase in Christensenellaceae, suggest poorer clinical outcomes. Although the changes in gut microbiota between the APS and PD groups were generally similar to those in the control group, there were notable exceptions. For instance, in MSA, Lachnospiraceae did not decrease, whereas Prevotellaceae showed a decline. In PSP, a reduction in the streptococcal family was observed.

The aforementioned changes in gut microbiota may influence the progression of APS through inflammatory mechanisms [176]. Research indicates that mutations in the gene encoding lysosomal glucocerebrosidase (GCase) represent the strongest genetic risk factor for DLB, with this gene believed to potentially induce DLB by triggering chronic neuroinflammation [177]. Regarding MSA, there is a growing body of evidence suggesting that a specific initial stimulus can lead to the misfolding of the aSyn protein, which in turn activates microglia and promotes the proliferation of astrocytes. Activated microglia can exhibit both pro-inflammatory and anti-inflammatory phenotypes, releasing specific inflammatory cytokines depending on their type, ultimately resulting in an inflammatory response and the onset of MSA [178,179,180,181,182]. Furthermore, the mechanisms by which the pathological protein tau contributes to PSP are gradually being elucidated. Some studies propose that the accumulation of phosphorylated tau promotes microglial activation, leading to neuroinflammation and facilitating the development of PSP. Additionally, an increase in double-stranded RNA levels in astrocytes has been observed in PSP, which is also linked to inflammatory responses [183,184,185,186]. Finally, the relationship between inflammation and CBS has been substantiated through its correlation with infection. In cases of neurosyphilis, some researchers have classified its clinical presentation as CBD, noting that neurosyphilis is closely associated with inflammatory responses, thereby indirectly confirming the connection between CBD and inflammation [187,188].

The evidence presented indicates that neuroinflammation serves as a primary driving factor in APS, suggesting that inflammation may represent a viable therapeutic target for modifying the disease process. Previous studies have explored the treatment of APS by modifying gut microbiota composition to influence the inflammatory response or by administering anti-inflammatory medications. Currently, for the treatment of MSA, drugs such as CSF1R selective inhibitors (e.g., PLX5622) and minocycline are predominantly utilized, with a focus on inflammation as a potential therapeutic target. MSA treatment aims to inhibit microglial activation and mitigate inflammation [189]. Additionally, there are ongoing investigations into the treatment of PSP, including strategies to alter gut microbiota composition through fecal transplantation and the use of nonsteroidal anti-inflammatory drugs to reduce inflammatory stimuli, thereby potentially modifying the progression of PSP [190,191,192].

## 8. Summary and Outlook

The connection between gut microbiota dysbiosis and PD primarily operates through the gut–brain axis. This axis consists of two anatomical routes: nerves and bodily fluids, enabling two-way communication between the gut and brain through signals from inflammatory cytokines. Dysbiosis leads to modifications in the makeup of metabolites produced by gut microbiota, potentially weakening barrier integrity and changing inflammation levels. These changes are significant in the relay of inflammatory factor signals within the gut–brain axis. Ultimately, these metabolites affect the progression of PD by altering how inflammatory signals are transmitted in this pathway.

The growing body of research on the association between gut microbiota, inflammation, and PD has contributed to a more comprehensive and systematic understanding of the underlying mechanisms and treatment methods in this field. However, this progress also highlights several challenges that warrant reflection. Firstly, some studies have established causal relationships among relevant factors without exploring the underlying mechanisms, such as the specific ways in which metabolites influence PD, the connection between gut microbiota and PD, and the precise microbial populations responsible for metabolite production. Secondly, many investigations rely on animal models, which may limit the applicability of findings to human patients due to genetic differences between species. Furthermore, some studies fail to account for confounding factors, including the microbial control levels of laboratory animals, methods of administration, the use of single versus combination therapies, drug concentration, disease duration, instrument bias, and demographic variables such as race, all of which can impact experimental outcomes. In addition, a common limitation across research in this area is the insufficient consideration of genetic variations within specific populations. Lastly, the use of cross-sectional research designs in some experiments restricts the ability to establish causal relationships between variables. Future research should address these limitations to yield more comprehensive and applicable results.

As technologies like multi-omics analysis and animal studies have progressed, we have been capable of identifying metabolites from gut microbiota in individuals with PD and revealing further underlying mechanisms. However, these techniques have certain limitations; for instance, metabolomics, which has limited coverage, is still relatively new, and some animal experiments face ethical concerns that restrict their widespread application. Consequently, researchers must further develop innovative methods to investigate the inflammatory mechanisms associated with gut microbiota metabolites in PD through technological advancements.

Mechanistic studies on PD, focusing on gut microbiota metabolites, have emerged, highlighting various treatment approaches that involve the regulation of these metabolites, including dietary interventions, probiotics, fecal transplantation, and the treatment methods based on comorbidity patterns. Clinical experiments have demonstrated positive effects from these methods, prompting researchers to investigate further potential benefits. However, these approaches remain in their early stages and are constrained by limitations related to the target population and implementation strategies, particularly given the ethical concerns surrounding fecal transplantation. To progress, it is crucial to tackle these challenges and create further approaches centered on metabolites from gut microbiota in order to enhance associated research.

## Abbreviations

The following abbreviations are used in this manuscript:

PD	Parkinson’s disease
α-syn	α-synuclein
SCFAs	short-chain fatty acids
BBB	blood-brain barrier
CNS	central nervous system
HPA	hypothalamic–pituitary–adrenal axis
KYNA	Kynurenic acid
AA	anthranilic acid
QA	quinolinic acid
5-HT	5-hydroxytryptamine
NMDA	N-methyl-D-aspartate
HFD	high-fat diet
Bas	bile acids
TGR5	Takeda G-protein-coupled receptor 5
TUDCA	tauroursodeoxycholic acid
FXR	farnesoid X receptor
DCs	dendritic cells
SMVT	sodium-dependent multivitamin transporter
BCAAs	Branched-Chain Amino Acids
GABA	gamma-aminobutyric acid
LC	Liquid Chromatography
GC	Gas Chromatography
NMR	Nuclear Magnetic Resonance
MS	Mass Spectrometry
PGA	pyroglutamate
NMS	non-motor symptoms
PD-MCT	PD multimodal comprehensive therapy
SIBO	small intestinal bacterial overgrowth
**ω**-3 FAs	omega-3 fatty acids
CI	cognitive impairment
DBS	deep-brain stimulation
APS	atypical Parkinson’s syndrome
DLB	Lewy body dementia
PSP	progressive supranuclear palsy
MSA	multiple system atrophy
CBD	cortical basal ganglia degeneration
